# Deletion of IκB‐Kinase β in Smooth Muscle Cells Induces Vascular Calcification Through β‐Catenin–Runt‐Related Transcription Factor 2 Signaling

**DOI:** 10.1161/JAHA.117.007405

**Published:** 2018-01-04

**Authors:** Isehaq Al‐Huseini, Noboru Ashida, Takeshi Kimura

**Affiliations:** ^1^ Department of Cardiovascular Medicine Graduate School of Medicine Kyoto University Kyoto Japan

**Keywords:** atherosclerosis, IKKβ, nuclear factor‐κB, vascular biology, vascular calcification

## Abstract

**Background:**

Vascular calcification was previously considered as an advanced phase of atherosclerosis; however, recent studies have indicated that such calcification can appear in different situations. Nevertheless, there has been a lack of mechanistic insight to explain the difference. For example, the roles of nuclear factor‐κB, a major regulator of inflammation, in vascular calcification are poorly explored, although its roles in atherosclerosis were well documented. Herein, we investigated the roles of nuclear factor‐κB signaling in vascular calcification.

**Methods and Results:**

We produced mice with deletion of IKKβ, an essential kinase for nuclear factor‐κB activation, in vascular smooth muscle cells (VSMCs; KO mice) and subjected them to the CaCl_2_‐induced aorta injury model. Unexpectedly, KO mice showed more calcification of the aorta than their wild‐type littermates, despite the former's suppressed nuclear factor‐κB activity. Cultured VSMCs from the aorta of KO mice also showed significant calcification in vitro. In the molecular analysis, we found that Runt‐related transcription factor 2, a transcriptional factor accelerating bone formation, was upregulated in cultured VSMCs from KO mice, and its regulator β‐catenin was more activated with suppressed ubiquitination in KO VSMCs. Furthermore, we examined VSMCs from mice in which kinase‐active or kinase‐dead IKKβ was overexpressed in VSMCs. We found that kinase‐independent function of IKKβ is involved in suppression of calcification via inactivation of β‐catenin, which leads to suppression of Runt‐related transcription factor 2 and osteoblast marker genes.

**Conclusions:**

IKKβ negatively regulates VSMC calcification through β‐catenin–Runt‐related transcription factor 2 signaling, which revealed a novel function of IKKβ on vascular calcification.

**Clinical Perspective**

**What Is New?**

- Inhibition of IKKβ, an essential kinase for nuclear factor‐κB activation, in vascular smooth muscle cells, augments vascular calcification.
- IKKβ has a kinase‐independent function, and it is involved in the suppression of vascular calcification.

**What Are the Clinical Implications?**

- IKKβ is the target of aspirin, and statins suppressed the activity of nuclear factor‐κB, but both are reported to have no suppressing effect. They even accelerate vascular calcification.
- This study could account for the mechanism, at least in part.

## Introduction

Vascular calcification was previously considered as a degenerative process seen in advanced atherosclerosis lesions, but recent studies have indicated that such calcification can appear in a different manner. Calcification not only accompanies with atherosclerosis but also accompanies diabetes mellitus or renal failure in the absence of atherosclerosis.^1^, ^2^ Moreover, recent clinical studies have revealed that statins, a medicine widely used for atherosclerosis, promote coronary calcification,^3^, ^4^, ^5^, ^6^, ^7^, ^8^ which clearly indicates that calcification stands on a different basis from atherosclerosis.^9^ However, the difference has not been fully explained because the mechanism of vascular calcification is poorly understood. For example, the roles of nuclear factor‐κB (NF‐κB) in the formation and progression of calcification have not been adequately assessed, although its roles in atherosclerosis have been well studied.^10^, ^11^

NF‐κB is a family of transcription factors that plays critical roles in inflammation and atherosclerosis, and reports have indicated that it is a target of statins.^12^, ^13^ NF‐κB generally exists as a homodimer or heterodimer in the cytosol that is bound to the inhibitor of κB (IκB).^14^ In response to a wide variety of stimuli, including inflammatory cytokines, IκB is phosphorylated and degraded via the ubiquitin pathway, which is followed by NF‐κB translocation to the nucleus and activation of transcription. The serine phosphorylation of IκB is mediated by a large multiunit complex containing 2 catalytic subunits, IKKα and IKKβ, and the regulatory subunit IKKγ or NF‐κB essential modulator (NEMO).^15^ Of these subunits, IKKβ is the essential kinase that mediates IκB phosphorylation.^15^

In this study, we produced mice with a deletion of IKKβ in vascular smooth muscle cells (VSMCs), a cell type contributing to calcification,^16^ and subjected them to CaCl_2_‐mediated aortic injury, which is an established animal model for vascular calcification.^17^, ^18^ Unexpectedly, we observed more calcification in the aorta of IKKβ‐deleted mice and found the activation of β‐catenin and Runt‐related transcription factor 2 (Runx2) as a possible mechanism. Furthermore, we present, for the first time, that the kinase‐independent function of IKKβ has an effect on the inactivation of β‐catenin, which leads to suppressing the calcification of VSMCs. These results indicate the protective effect of IKKβ on vascular calcification, which at least partly explains the difference between the smooth muscle calcification and atherosclerosis observed in clinical settings.

## Methods

The data, analytic methods, and study materials will not be made available to other researchers for purposes of reproducing the results or replicating the procedure because some materials are used for other unpublished projects.

### Mice

All animal experiments were performed in accordance with the institutional guidelines of the Institute of Laboratory Animals, Graduate School of Medicine, Kyoto University (Kyoto, Japan). For this study, transgenic mice were crossed onto a C57BL/6J background >10 times. To block smooth muscle cell NF‐κB signaling, we used IKKβ knockout selectively in smooth muscle cells of mice. IKKβ flox/flox mice were generously given by Professor Michael Karin and mated with Sm22α, a protein that is specifically expressed in smooth muscle cells, and Cre^+^ mice (The Jackson Laboratory, Bar Harbor, ME) to generate IKKβ flox/wt Sm22α‐Cre^+/−^ mice, which were mated with IKKβ flox/flox mice to generate IKKβ flox/flox Sm22α‐Cre^+/−^ (KO) mice or IKKβ flox/flox Sm22α‐Cre^−/−^ (wild‐type [WT]) mice. To generate transgenic mice with constitutively active IKKβ kinase (KA)^19^ selectively in smooth muscle cells, IKKβ‐KA flox/flox mice were mated with Sm22α‐Cree^+/−^ mice to generate IKKβ‐KA flox/wt Sm22α‐Cre^+/−^ (KA) mice or IKKβ‐KA flox/wt Sm22α‐Cre^−/−^ (WT) mice. To produce transgenic mice with kinase‐dead IKKβ (KD) in smooth muscle cells, IKKβ‐KD flox/flox mice were mated with IKKβ flox/flox Sm22α‐Cre^+/−^ (KO) to generate IKKβ‐KD flox/flox IKKβ flox/flox Sm22α‐Cre^+/−^ (KD) mice. These KD mice have complete knockout of endogenous IKKβ from VSMCs, followed by knockin of kinase defective form of IKKβ. In the mating protocol for all kinds of mice, we could have WT mice within littermate; therefore, we used the littermate WT as control mice in all experiments.

### Generation of IKKβ (K44A) (KDIKKβ flox/flox) Knock‐In Mice

To generate conditional IKKβ (K44A) expressing mice, a CAG promoter, a loxP flanked STOP cassette, and

IKKβ (K44A) cDNA were inserted into the mouse Gt(Rosa)26Sor locus by homologous recombination. A 3.4‐kb upstream DNA fragment and a 4.3‐kb downstream DNA fragment from the unique XbaI site in intron 1 of Gt(Rosa)26Sor locus were amplified by polymerase chain reaction (PCR) from RENKA ES cell genomic DNA and used as the homology arms for the targeting vector. To construct the expression unit, a chemically synthesized DNA sequence containing a DYKDDDDK‐tagged mouse IKKβ (K44A), a rabbit β‐globin polyadenylation signal, and a VloxP sequence were combined with an frt‐flanked PGK_neo cassette (phosphoglycerate kinase 1 promoter‐driven neomycin‐resistant gene). At the 5′ region of this expression unit, a CAG promoter and a loxP flanked STOP cassette containing chloramphenicol acetyltransferase cDNA and Vlox43L were inserted. Then, the promoter‐expression unit was subcloned between 2 homology arms. As a negative selection marker, an MC1 promoter‐driven diphtheria toxin A cassette was placed at the 5′ region of the homology arm. The resulting knock‐in targeting vector contains MC1 promoter‐driven diphtheria toxin A, a 3.4‐kb 5′ homologous arm, a CAG promoter, the first loxP site, chloramphenicol acetyltransferase cDNA, polyA, Vlox43L, an frt‐flanked PGK_neo cassette, the second loxP site, a DYKDDDDK tag, IKK2 (K44A) cDNA, bGH polyA, VloxP, and a 4.3‐kb 3′ homology arm. This vector was linearized and introduced into RENKA ES cells (C57BL/6 genetic background) by electroporation. After selection using geneticin, the resistant clones were isolated and their DNA was analyzed to identify homologous recombinants by PCR using the following primer sets: sc_R10603, 5′‐GTTACTCCACTTTCAAGTTCCTTATAAGC‐3′; neo_108r, 5′‐CCTCAGAAGAACTCGTCAAGAAG‐3′; sc_5AF3, 5′‐ATCCTAAGGTCACTTTTAAATTGAGG‐3′; and neo_G02, 5′‐ATCAGGACATAGCGTTGGCTAC‐3′. Genomic Southern hybridization was probed with a neomycin resistance gene. Homologous recombinant ES cell clones were aggregated with 8‐cell embryos of ICR mice to generate chimeric mice. Chimeric mice with a high contribution from the RENKA background were bred with C57BL/6 mice. Germline transmitted mice were mated to B6;D2‐Tg(CAG‐FLP)18Imeg mice to eliminate the PGK_neo cassette from the genome through Flp/frt‐mediated excision. The knock‐in allele was identified by PCR with the following primers: 5AF1, 5′‐CCATTGGCTCGTGTTCGTG‐3′; and mutant_R, 5′‐CCGGGGGATCCACTAGTA‐3′. The WT allele was identified by PCR with the following primers: 5AF1 and wild_R, 5′‐ACTCCCGCCCATCTTCTAGA‐3′.

### CaCl_2_‐Mediated Abdominal Aorta Injury

An abdominal aorta injury was induced in 8‐ to 14‐week‐old KO and WT male mice by periaortic application of CaCl_2_, as described previously,^17^, ^18^ with minor modifications. We started with a conventional protocol of 20 minutes CaCl_2_ treatment, but too much dilatation and calcification seen in WT aorta interfere with the evaluation of the difference between WT and KO aorta. Therefore, we reduced the CaCl_2_ incubation period to 10 minutes. Even in that protocol, 2 weeks after CaCl_2_ application, the aortas in both types of mice were significantly dilated when compared with those in the sham–operated on mice. Mice were anesthetized by IP injection with pentobarbital (100 mg/kg) and then underwent a laparotomy. An incision of ≈2 cm was made, and the small and large intestines were moved away to expose the infrarenal aorta. Small pieces of gauzes soaked with 0.6 mol/L CaCl_2_ were placed circumferentially on the infrarenal aorta for 10 minutes. Then, the gauze was removed and the aorta was rinsed with 0.95% NaCl and wiped with a cotton swab. NaCl (0.95%) was substituted for CaCl_2_ in sham–operated on mice. The incision was closed, and mice were placed on a warm plate until they had recovered from anesthesia. After the surgical procedure, the animals were allowed free access to food and water until the day of euthanasia.

### Aorta Collection and Size Measurement

Two weeks after the operation, mice were euthanized with an overdose of pentobarbital. The vascular system was flushed with cold PBS from the left ventricle. The abdominal aorta was carefully dissected under a microscope, cleaned of extraneous tissue, and then photographed for diameter measurement.

### Histology and Immunohistochemistry

Approximately 2 to 3 mm of infrarenal aorta was fixed with 4% paraformaldehyde overnight, embedded in paraffin, cut into 4‐μm cross sections. Then, cross sections were deparaffinized 3 times in xylene and dehydrated in 80%, 90%, and 100% ethanol for 5 minutes each. Elastin was visualized using the fluorescence properties of hematoxylin and eosin.^20^ Alizarin Red staining was performed to detect calcification for both the in vivo and in vitro samples. For immunohistochemical staining, cross sections were boiled in 10 mmol/L citrate buffer for antigen retrieval, followed by avidin/biotin. Sections were then incubated with primary antibodies that recognize SM22α, IKKβ, Runx2, and active β‐catenin overnight at 4°C. The secondary antibody IgG/biotin goat anti‐rabbit or goat anti‐mouse was incubated for 1 hour at room temperature, followed by diaminobenzidine. Sections were visualized using a BIOREVO BZ‐9000 Keyence microscope.

### Cell Culture

VSMCs from the aorta of littermate mice were isolated on the basis of a protocol described by Villa‐Bellosta and Hamczyk.^21^ Briefly, aortas were perfused with cold PBS. The aorta from the aortic arch to the iliac bifurcation was isolated and incubated in collagenase type 2 solution for 15 minutes at 37°C in 5% CO_2_. Then, adventitia was rolled off the medial layer. Medial layers were minced and further incubated for 2 hours at 37°C with agitation. Resuspended cells were centrifuged for 5 minutes at 300*g*. The supernatant was aspirated, and the cell pellet was resuspended in an appropriate volume of DMEM supplemented with 10% fetal bovine serum (FBS). Finally, cell suspensions were cultured in collagenase type 1 coated culture dishes and maintained in DMEM supplemented with 10% FBS, 100 U/mL of penicillin, and 100 μg/mL of streptomycin in a 5% CO_2_/water‐saturated incubator at 37°C. Each kind of VSMCs was isolated from 1 mouse of KO, KA, KD, and littermate WT, and individual samples that used in vitro experiments were harvested from separately cultured dishes of cells.

### Transient Transfection

KO VSMCs were transfected with 1 μg of IKKβ‐KD or IKKβ‐KA plasmids using transfection reagent Effectene (Qiagen, Valencia, CA), according to the manufacturer's instructions. Two days after transfection, VSMCs were harvested and then used in Western blotting. Plasmids encoding IKK‐2 K44M (IKKβ‐KD) and IKK‐2 S177E S181E (IKKβ‐KA) were from Anjana Rao Laboratory and procured through Addgene (Addgene plasmid no. 11104 and no. 11105, respectively).^19^

### Western Blotting

VSMCs were harvested from culture dishes using cell scraper tools. Protein extraction was performed on ice and using a cell lysis buffer (9803S; Cell Signaling) with a protease inhibitor cocktail. After the total protein concentration was measured, the same amount of extracted protein was loaded for SDS‐PAGE immunoblot analysis and transferred to nitrocellulose membranes. Next, membranes were blocked with 5% nonfat milk or 5% BSA in Tris buffer solution containing 0.05% Tween‐20 for 1 hour at room temperature, with gentle agitation. The membrane was immunoblotted with primary antibody overnight at 4°C. The membrane was washed 3 times with Tris buffer solution containing 0.05% Tween‐20 for 10 minutes each. This was followed by a 1‐hour incubation with a horseradish peroxidase–conjugated secondary antibody. Blots were visualized using the ECL Western Blotting Detection kit (GE Healthcare). Each band was normalized by the corresponding value of β‐actin or GAPDH as an internal control. Densitometric analysis was performed by ImageJ Software. All experiments were performed at least in triplicate.

### Quantitative Real‐Time PCR

Total RNA was extracted from cultured VSMCs using an RNAqueous total RNA isolation kit (AM1912; Thermo Fisher Scientific K.K., Yokoyama, Japan) according to the manufacturer's instructions. cDNA was generated from total RNA using The High Capacity RNA‐to‐cDNA Kit (4387406). Quantitative PCR was performed using the QuantiTect SYBR Green PCR Kit and real‐time PCR system (StepOnePlus; Applied Biosystems Japan, Tokyo, Japan). Relative mRNA level of ostrix, alkaline phosphatase, and osteocalcin was normalized to the Rn18s mRNA level in the same sample.

### Antibodies and Reagents

Antibodies used include anti‐IKKβ (Millipore, Billerica, MA), anti‐Transgelin (for SM22α; Lifespan Bioscience, Seattle, WA), anti‐GAPDH, Runx2, anti‐mouse IgG horseradish peroxidase–linked antibody and anti‐rabbit IgG horseradish peroxidase–linked antibody (CST), active β‐catenin, total β‐catenin (Santa Cruz, Dallas, TX), anti‐p65 (Santa Cruz), monoubiquitinylated and polyubiquitinylated conjugate monoclonal antibody (ENZO), goat anti‐rabbit IgG, Alexa Fluor 488 goat anti‐mouse IgG, Alexa Fluor 594 donkey anti‐goat IgG, and Alexa Fluor 488 donkey anti‐goat IgG (Invitrogen, Foster City, CA). The reagents used included interleukin‐1β (Wako Pure Chemical Industries, Ltd, Osaka, Japan) and NE‐PER Nuclear and Cytoplasmic Extraction Reagents (Thermo).

### Statistical Analysis

Experimental results are expressed as the mean±SD. Groups were compared using the 2‐tailed Student *t* test or 1‐ and 2‐way ANOVA, followed by the Tukey test. *P*<0.05 was considered statistically significant.

## Results

### Generation of Mice With Deletion of IKKβ in VSMCs

**Figure 1:**
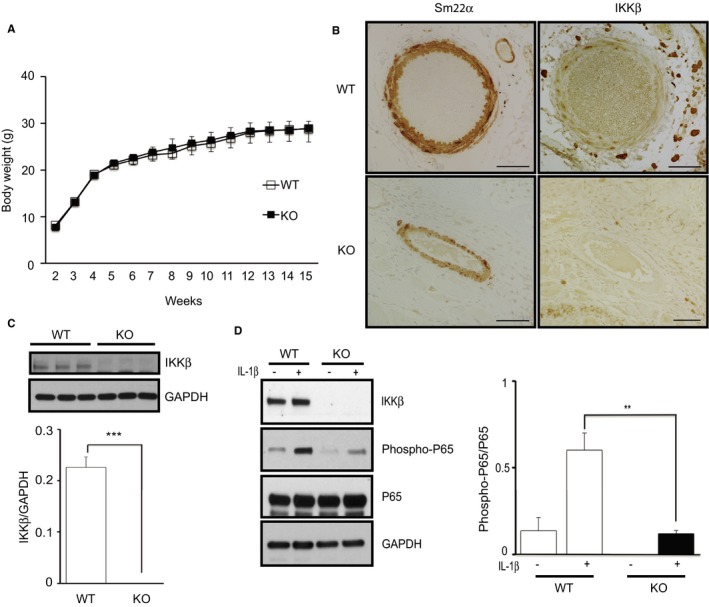
Making mice with deletion of IKKβ in vascular smooth muscle cells (VSMCs). A, Body weight±SD of wild‐type (WT) and IKKβ knockout (KO) mice at the indicated age (n=5). B, Representative images of immunostaining of the aorta section with anti‐SM22α and anti‐IKKβ antibodies. Bar=50 μm. C, Representative Western blots and densitometric analysis of IKKβ expression in cultured VSMCs isolated from WT and KO littermate mice. Data represent the mean±SD (*t* test, n=3). D, Representative Western blots and densitometric analysis of phosphorylated p65/total p65 in cultured VSMCs with stimulation by interleukin (IL)‐1β (2.5 ng/mL) or the vehicle for 15 minutes. Data represent the mean±SD (2‐way ANOVA, n=3). **P<0.01, ***P<0.001.

To inactivate NF‐κB signaling in VSMCs, we produce mice with a deletion of IKKβ selectively in smooth muscle cells (KO mice), as previously described. Mice were born according to mendelian frequency, and there was no significant difference in growth between WT and KO mice (Figure 1A). Immunohistochemistry analyses indicated that IKKβ was successfully knocked out in SM22α‐positive VSMCs in IKKβ knockout mice (Figure 1B). Furthermore, we performed cultured VSMCs from the aorta of KO and littermate control mice and confirmed that IKKβ is deleted in VSMCs of KO mice (Figure 1C).

### Suppression of NF‐κB Activity in IKKβ‐Deficient VSMCs

Next, we examined the NF‐κB activity in IKKβ‐deficient VSMCs. IKKβ activates NF‐κB via the phosphorylation and degradation of IκB and eventually phosphorylates p65, a major subunit of NF‐κB.^15^ VSMCs of the aorta from KO and control mice were stimulated by interleukin‐1β to activate NF‐κB signaling, and we found that the phosphorylation of p65 was significantly suppressed in KO cells (Figure 1D). These results confirm that NF‐κB is suppressed in VSMCs from KO mice.

### Deletion of IKKβ in VSMCs Promotes Aortic Calcification in CaCl_2_‐Induced Abdominal Aorta Injury

**Figure 2:**
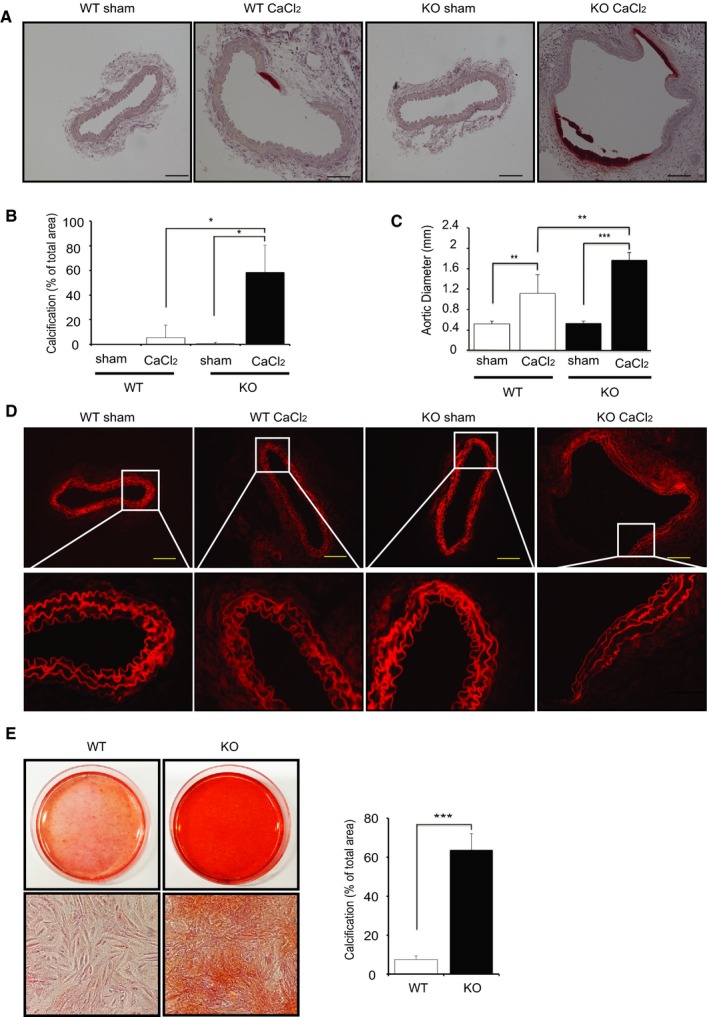
Deletion of IKKβ in smooth muscle cells promotes calcification. A, Representative microscopy images of cross sections of infrarenal aortas stained with Alizarin Red from wild‐type (WT) and IKKβ knockout (KO) littermate mice 2 weeks after saline (sham) or CaCl_2_ treatment. B, Quantification of vascular calcification. Calcification was quantified by ImageJ software. Graph presented is the percentage of positively stained medial layer areas in the total area of medial layer. Bars represent the mean±SD (2‐way ANOVA, n=5). C, Graph is the maximal external diameter of the WT and KO infrarenal aorta 2 weeks after saline (sham) or CaCl_2_ treatment. Bars represent the mean±SD (2‐way ANOVA, n=5). D, Representative fluorescence microscopy images of medial elastic lamellae in the cross sections of infrarenal aortas 2 weeks after saline (sham) or CaCl_2_ treatment. E, Representative microscopy images of Alizarin Red staining of 4‐week cultured vascular smooth muscle cells (VSMCs) isolated from WT and KO littermate mice. Calcification was quantified by ImageJ software. Graph presented is the percentage of positively stained area in the total area randomly selected. VSMCs were cultured in normal medium with 10% fetal bovine serum. Bars represent the mean±SD (*t* test, n=6). *P<0.05, **P<0.01, ***P<0.001.

To elucidate the roles of IKKβ/NF‐κB signaling in vascular calcification, we subjected WT and KO mice to a CaCl_2_‐induced aorta injury model. Unexpectedly, KO mice showed more significant calcification in aorta, and those aortas were more dilated compared with WT mice (Figure 2A through 2C). Histologically, the aorta of KO mice revealed extensive damage in the medial layer and more disruption and elongation of the medial elastic lamellae, possibly because of the significant calcification (Figure 2D). Consistent with these in vivo results, Alizarin Red staining of 4‐week cultured VSMCs showed remarkable calcium deposition in KO VSMCs but not in WT VSMCs (Figure 2E).

### Deletion of IKKβ Induces Transdifferentiation of VSMCs to Osteoblast‐Like Cells via Activating the β‐Catenin–Runx2 Signaling Pathway

**Figure 3:**
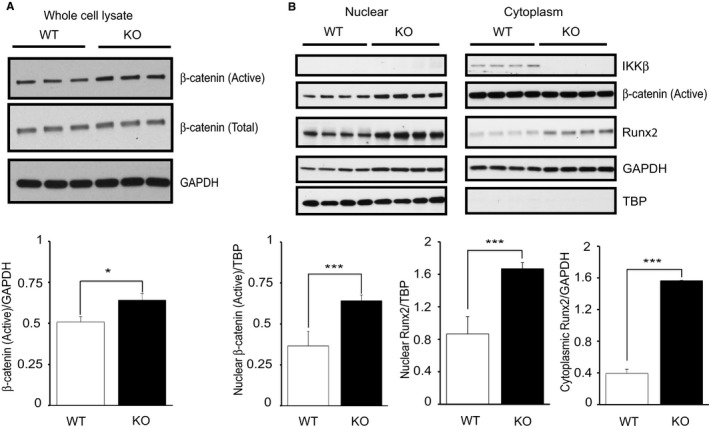
Activated β‐catenin and Runt‐related transcription factor 2 (Runx2) in IKKβ‐deficient vascular smooth muscle cells (VSMCs). A, Representative Western blots and densitometric analysis of active β‐catenin/GAPDH in a whole cell lysate of cultured VSMCs isolated from wild‐type (WT) and IKKβ knockout (KO) littermate mice. Bars represent the mean±SD (*t* test, n=3). B, Representative Western blots and densitometric analysis of Runx2 and β‐catenin expression in nuclear and cytoplasmic extractions normalized to tata‐binding protein (TBP) or GAPDH, respectively. Bars represent the mean±SD (*t* test, n=4). *P<0.05, ***P<0.001.

To determine the reason why injured aortic wall and cultured VSMCs of KO mice showed more calcium deposition, we investigated the underlying molecular mechanism. Recently, it is widely accepted that pathways controlling vascular calcification and bone remodeling are similar.^22^, ^23^ Several studies have shown that the activation of β‐catenin, which is the prototypic mediator and marker of canonical Wnt signaling, is necessary for osteoblast formation and is involved in the calcification of VSMCs by promoting the expression of osteocyte genes, which leads to the transdifferentiation of VSMCs to osteoblast‐like cells.^24^, ^25^ Therefore, we investigated the activity of β‐catenin in cultured VSMCs and found that the expression of the nonphosphorylated active form of β‐catenin in the whole cell lysate of VSMCs was significantly augmented in KO cells when compared with that in WT cells (Figure 3A). We also assessed active β‐catenin in nuclear extraction to confirm its translocation to the nucleus for osteocyte gene expression and found that more active β‐catenin was located in the nucleus of KO VSMCs (Figure 3B). Next, we examined Runx2 expression in cultured VSMCs. Runx2 is a major transcription factor playing a crucial role in VSMC transdifferentiation into osteoblast‐like cells^26^, ^27^ by regulating osteoblast genes, and it is regulated by β‐catenin.^24^ Consistent with the β‐catenin results, the expression of Runx2 was significantly more increased in KO VSMCs compared with that in WT VSMCs in both nucleus and cytoplasm (Figure 3B). These results indicate that the ablation of IKKβ in VSMCs accelerates calcification through upregulation of β‐catenin and Runx2 signaling.

### Constitutively Active IKKβ Protects VSMCs From Calcification via the Downregulation of β‐Catenin and Runx2 Expression

**Figure 4:**
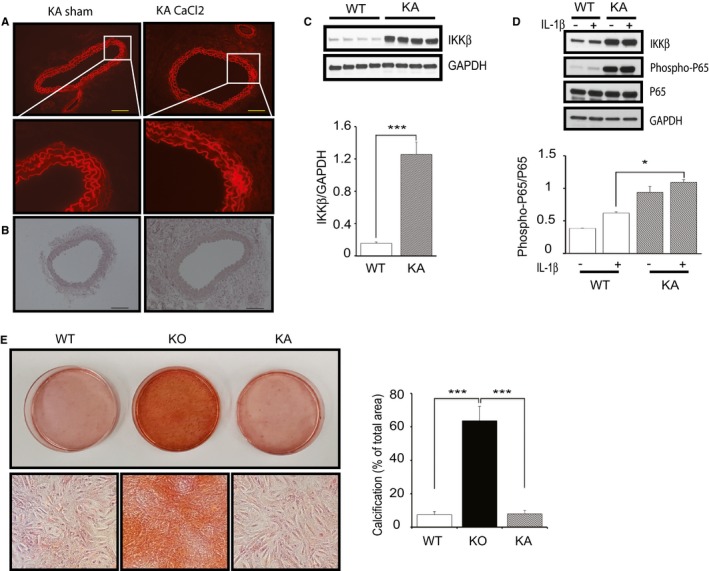
Constitutive activation of IKKβ in vascular smooth muscle cells (VSMCs) inhibits calcification. A, Representative fluorescence microscopy images of medial elastic lamellae in the cross sections of infrarenal renal aortas from kinase‐active IKKβ (KA) mice 2 weeks after saline (sham) or CaCl_2_ treatment. Bar=50 μm. B, Representative microscopy images of cross sections of infrarenal aortas stained with Alizarin Red from KA mice 2 weeks after saline (sham) or CaCl_2_ treatment. Bar=50 μm. C, Representative Western blots and densitometric analysis of IKKβ/GAPDH expression in cultured VSMCs isolated from wild‐type (WT) and KA littermate mice. Bars represent the mean±SD (*t* test, n=4). D, Representative Western blots and densitometric analysis of phosphorylated p65/total p65 expression in cultured VSMCs isolated from WT and KA littermate mice with stimulation by interleukin (IL)‐1β (2.5 ng/mL) or the vehicle for 15 minutes. Bars represent the mean±SD (2‐way ANOVA, n=3). E, Representative microscopy images of Alizarin Red staining of 4‐week cultured VSMCs isolated from WT, IKKβ knockout (KO), and KA mice and quantification of VSMC calcification. VSMCs were cultured in normal medium with 10% fetal bovine serum. Calcification was quantified by ImageJ software. Graph presented is the percentage of positively stained area in the total area randomly selected. Bars represent the mean±SD (1‐way anova, n=6). *P<0.05, ***P<0.001.

**Figure 5:**
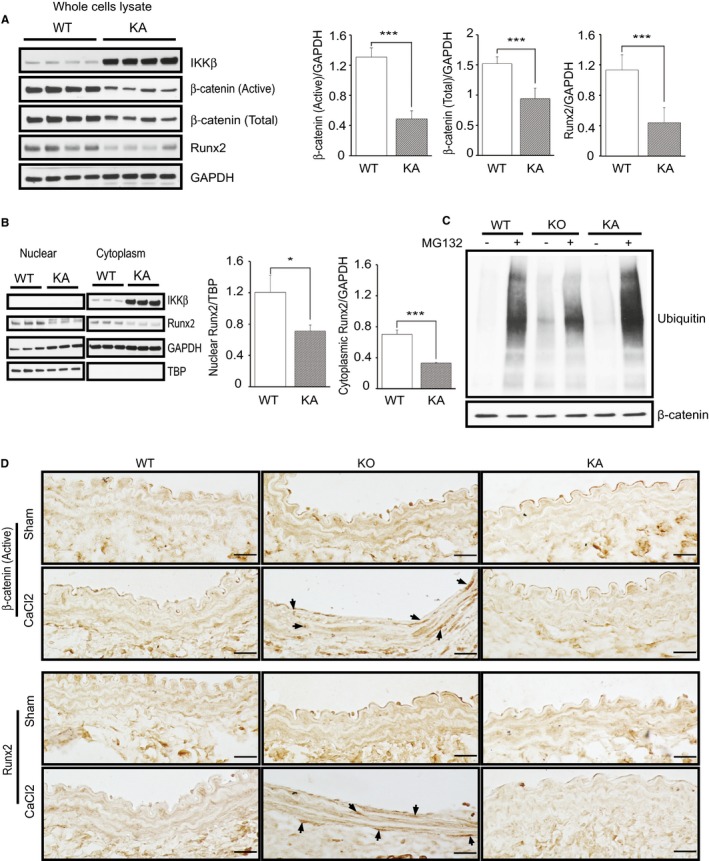
Constitutive activation of IKKβ in vascular smooth muscle cells (VSMCs) inhibits β‐catenin–Runt‐related transcription factor 2 (Runx2) signaling. A, Representative Western blots and densitometric analysis of active and total β‐catenin/GAPDH and Runx2/GAPDH expression in a whole cell lysate of cultured VSMCs isolated from wild‐type (WT) and kinase‐active IKKβ (KA) littermate mice. Bars represent the mean±SD (*t* test, n=4). B, Representative Western blots and densitometric analysis of Runx2 expression in nuclear and cytoplasmic extractions normalized to Tata‐binding protein (TBP) or GAPDH, respectively. Bars represent the mean±SD (*t* test, n=3). C, Representative image of 3 independent ubiquitination assays in which β‐catenin was precipitated and blotted with antiubiquitin. D, Representative images of immunostaining of the aorta section from WT, IKKβ knockout (KO), and KA mice 2 weeks after saline (sham) or CaCl_2_ treatment with antiactive β‐catenin and Runx2 antibodies. Bar=20 μm. *P<0.05, ***P<0.001.

To confirm the mechanism previously described, we examined the effect of kinase‐active IKKβ on vascular calcification as opposite situation. We produced mice with a knock‐in of kinase‐active IKKβ specifically in VSMCs (KA mice), as described previously,^19^ and subjected them to a CaCl_2_‐induced aorta injury model. Histologically, the aorta of KA mice showed an intact medial layer with negligible disruption in the medial elastic lamellae (Figure 4A), and no calcification was observed in the medial layer of the aorta after CaCl_2_ (Figure 4B). Western blot analysis of cultured VSMCs from KA mice confirmed the upregulation of IKKβ and phosphorylated p65 (Figure 4C and 4D), indicating the constitutive activation of NF‐κB in cultured VSMCs from KA mice. Consistent with in vivo results, Alizarin Red staining of 4‐week cultured KA VSMCs showed an absence of calcium deposits (Figure 4E). Next, we examined β‐catenin and Runx2 and found a significant decrease in both active and total β‐catenin and Runx2 in KA‐VSMCs when compared with that in WT (Figure 5A). Furthermore, nuclear location of Runx2 was significantly decreased in KA VSMCs (Figure 5B). These results indicated that overexpression of kinase‐active IKKβ negatively regulates β‐catenin–Runx2 signaling in KA VSMCs, which were opposite to the results seen in KO VSMCs.

### IKKβ Modulates Ubiquitination of β‐Catenin in VSMCs

According to previous reports, β‐catenin is inactivated by ubiquitination via the ubiquitin‐proteasome pathway after it is phosphorylated.^28^ Thus, we examined ubiquitination of β‐catenin on WT, KO, and KA‐VSMCs. Cells were pretreated with proteasome inhibitor MG132 to block ubiquitin‐induced degradation by proteasomes and subjected to ubiquitination assay. The results showed that the deletion of IKKβ in VSMCs reduced the ubiquitination of β‐catenin and, conversely, it was augmented in VSMCs with constitutively active IKKβ (Figure 5C). These results were consistent with the change of active β‐catenin in KO and KA VSMCs. We also histologically examined the change of β‐catenin and Runx2 in CaCl_2_‐injured aortas of WT, KO, and KA mice. As shown in Figure 5D, CaCl_2_ induces staining of active β‐catenin and Runx2 in nuclei of smooth muscle layers in KO aorta compared with that in sham group. In contrast, no apparent staining of active β‐catenin and Runx2 was observed in both KA and WT aorta, even after CaCl_2_‐induced aortic injury. These results are consistent with observations seen in cultured VSMCs.

### There is No Apparent Relationship Between Apoptosis and VSMC Calcification

**Figure 6:**
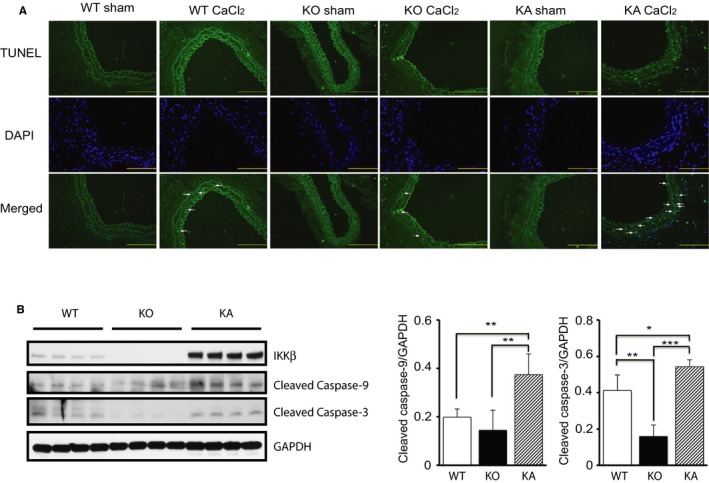
Apoptosis in injured aortas of IKKβ knockout (KO) and kinase‐active IKKβ (KA) mice. A, Representative images of terminal deoxynucleotidyl transferase dUTP nick end labeling (TUNEL) assay performed in cross sections of infrarenal aortas after 2 weeks CaCl_2_ or saline application in wild‐type (WT), KO, and KA mice. Bar=50 μm. B, Representative Western blots and densitometric analysis of cleaved caspase‐9 and caspase‐3/GAPDH expression in cultured vascular smooth muscle cells isolated from WT, KO, and KA mice. WT mice were from littermates of KO mouse. Bars represent the mean±SD (1‐way ANOVA, n=4). DAPI indicates 4′,6‐diamidino‐2‐phenylindole. *P<0.05, **P<0.01, ***P<0.001.

Several reports have shown that apoptosis of VSMCs can induce vascular calcification.^29^, ^30^ To investigate the involvement of apoptosis in VSMC calcification induced by IKKβ deletion, we performed terminal deoxynucleotidyl transferase dUTP nick end labeling staining on the aortas. There was no remarkable difference in number of apoptotic cells between WT and KO aortas after CaCl_2_ treatment. In contrast, terminal deoxynucleotidyl transferase dUTP nick end labeling staining revealed a remarkable increase of apoptotic cells after CaCl_2_‐induced aortic injury in KA mice, compared with WT and KO mice (Figure 6A). To understand the molecular mechanisms by which overexpression of kinase‐active IKKβ induced VSMC apoptosis, we examined caspase‐9 and caspase‐3, crucial mediators of apoptosis, in cultured VSMCs. Western blot analysis of cultured VSMCs demonstrated that expression of both cleaved caspase‐9 and cleaved caspase‐3 was significantly increased in KA VSMCs compared with those in WT and KO VSMCs (Figure 6B). Despite the increase of apoptosis at the medial layer of KA mice after CaCl_2_ application, no remarkable calcification was observed, as previously shown. Collectively, these observations suggest that the apoptosis is not the primary cause of VSMC calcification, at least in these mouse models.

### Kinase‐Independent Function of IKKβ Is Involved in Regulating VSMC Calcification

**Figure 7:**
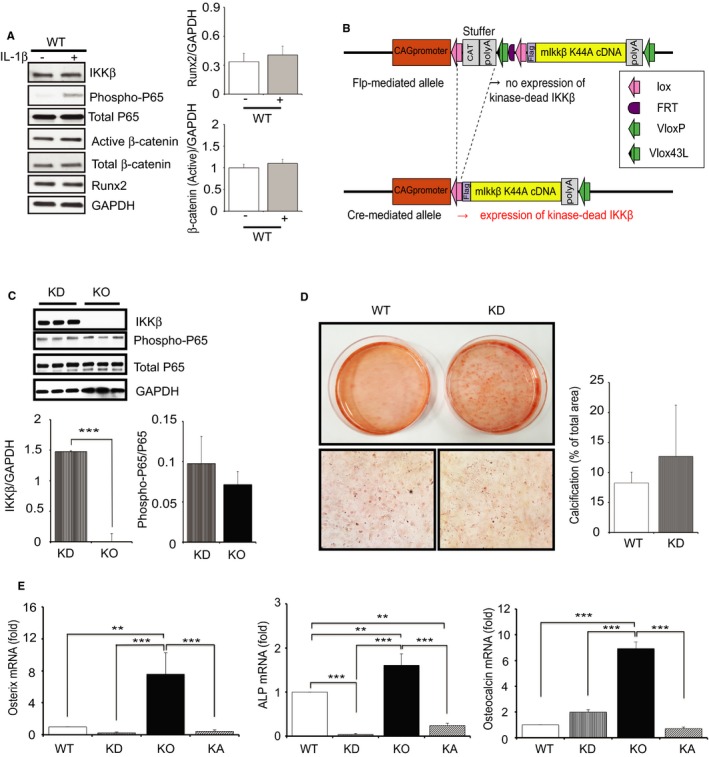
IKKβ regulates vascular smooth muscle cells (VSMC) calcification through its kinase‐independent function. A, Representative Western blots and densitometric analysis of active β‐catenin/GAPDH and Runt‐related transcription factor 2 (Runx2)/GAPDH expression in cultured VSMCs isolated from wild‐type (WT) mouse with stimulation by interleukin (IL)‐1β (2.5 ng/mL) or the vehicle for 15 minutes. Bars represent the mean±SD (*t* test, n=3). B, Kinase‐dead IKKβ transgene construct. C, Representative Western blots and densitometric analysis of IKKβ/GAPDH, phosphorylated p65/total p65 expression in VSMCs isolated from kinase dead (KD) and IKKβ knockout (KO) mice. Bars represent the mean±SD (*t* test, n=3). D, Representative microscopy images of Alizarin Red staining of 4‐week cultured VSMCs isolated from WT and KD mice and quantification of VSMC calcification. Calcification was quantified by ImageJ software. Graph presented is the percentage of positively stained area in the total area randomly selected. Bars represent the mean±SD (*t* test, n=6). E, Results of quantitative real‐time PCR (qRT‐PCR) for the expression of various osteogenic‐related genes (osterix, alkaline phosphatase [ALP], and osteocalcin) in WT, KD, KO, and kinase active IKKβ (KA) cells that were normalized to the Rn18s mRNA level. WT samples used in RT‐PCR were from littermate of KO mouse. Cells used in qRT‐PCR were cultured for 2 weeks in normal medium with 10% fetal bovine serum. Bars represent the mean±SD (1‐way ANOVA, n=4). **P<0.01, ***P<0.001.

Kinase‐dependent function of IKKβ in the activation of NF‐κB is well known; however, recent reports showed that IKKβ has other regulatory functions, independently from NF‐κB activation.^31^ Furthermore, we previously reported that IKKβ regulates other proteins, even in a kinase‐independent manner.^31^, ^32^ Results previously shown clearly indicated that IKKβ negatively regulates calcification, but it is difficult to conclude whether either or both of kinase‐dependent or kinase‐independent function of IKKβ is involved in it, as far as we compare among WT, KO, and KA‐IKKβ. Indeed, interleukin‐1β stimulation did not induce any change in Runx2 or β‐catenin in WT cells (Figure 7A), which implicates the possible involvement of kinase‐independent function of IKKβ in regulating these molecules.

To make it clear, we produced mice expressing kinase‐dead mutant (KD) of IKKβ in VSMCs on the background of IKKβf/f sm22Cre^+/−^ (KO) mice, according to the previous report showing that point mutation of an amino acid inactivates kinase function^19^ (Figure 7B), and we examined cultured VSMCs from KD mice. As shown in Figure 7C, IKKβ is highly more expressed in KD VSMCs compared with KO VSMCs. However, the level of phosphorylated p65 in KD cells is equivalent to that in KO cells, which indicated that the kinase activity is successfully lost in KD cells. Surprisingly, Alizarin Red staining showed no remarkable increase of calcium deposit in KD VSMCs compared with that in WT VSMCs (Figure 7D), contrary to KO VSMCs. Considering the significant calcification in KO VSMCs compared with that in WT VSMCs, it is clearly indicated that the kinase‐independent function of IKKβ has a suppressive effect on calcification. To confirm the difference in calcification among WT, KO, KA, and KD VSMCs, we performed real‐time PCR to examine the expression of major osteoblast marker genes regulating transdifferentiation of VSMCs to osteoblast‐like cells.^33^, ^34^ As shown in Figure 7E, osterix, alkaline phosphatase, and osteocalcin were significantly upregulated only in KO VSMCs.

**Figure 8:**
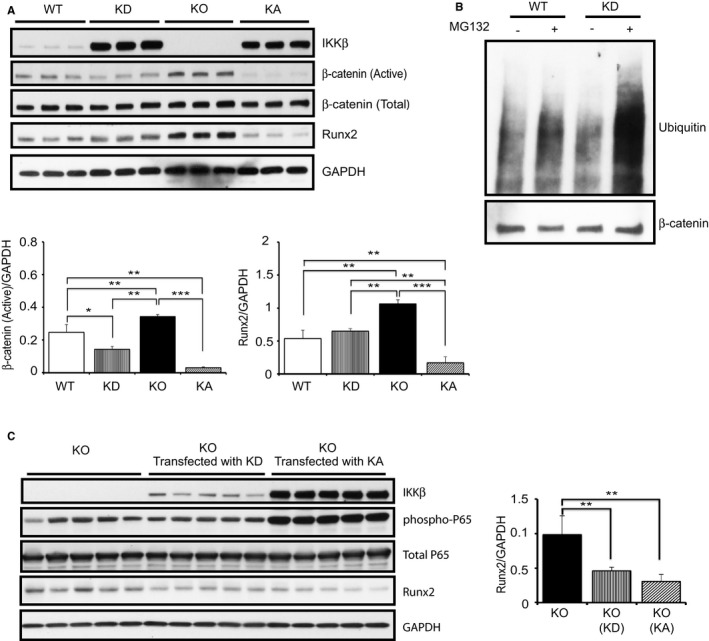
IKKβ inhibits β‐catenin–Runt‐related transcription factor 2 (Runx2) signaling through its kinase‐independent function. A, Representative Western blots and densitometric analysis of active β‐catenin/GAPDH and Runx2/GAPDH expression in vascular smooth muscle cells (VSMCs) isolated from wild‐type (WT), kinase dead (KD), IKKβ knockout (KO), and kinase active IKKβ (KA) mice. WT samples used in Western blot were from littermate of KO mouse. Bars represent the mean±SD (1‐way ANOVA, n=3). B, Representative image of 3 independent ubiquitination assays in WT and KD VSMCs, in which β‐catenin was precipitated and blotted with antiubiquitin. C, Representative Western blots and densitometric analysis of Runx2/GAPDH expression in KO VSMCs transfected with IKKβ‐KD and IKKβ‐KA plasmids. KO VSMCs were harvested after 2 days of transfection. Control KO VSMCs were transfected with no plasmid, but transfection reagent only. Bars represent the mean±SD (1‐way ANOVA, n=5). *P<0.05, **P<0.01, ***P<0.001.

Next, we examined β‐catenin and Runx2 signaling in KD VSMCs. Consistent with the previous results, active β‐catenin and Runx2 in KD VSMCs were significantly downregulated compared with KO VSMCs, clearly indicating that the kinase‐independent function of IKKβ is involved in the downregulation of β‐catenin (Figure 8A). We also noticed that active β‐catenin and Runx2 in KD VSMCs were significantly more expressed than those in KA VSMCs, despite more expression of IKKβ in KD cells, which indicated that kinase‐dependent function of IKKβ also has a suppressive effect on calcification. Furthermore, we performed β‐catenin ubiquitination assay in KD VSMCs and found that β‐catenin ubiquitination was upregulated in KD VSMCs (Figure 8B), which supported the observation that β‐catenin is inactivated in KD VSMCs. To confirm the observations further, KO VSMCs were transfected with IKKβ‐KD and IKKβ‐KA plasmids. KO VSMCs transfected with IKKβ‐KD or IKKβ‐KA plasmids showed a significant decrease in Runx2 expression (Figure 8C), which is consistent with results seen in transgenic VSMCs.

## Discussion

Recent clinical studies have indicated that vascular calcification is different from atherosclerosis, but the mechanisms of vascular calcification have not been explored adequately. In this study, we show that the deletion of IKKβ, an essential kinase for NF‐κB activation, in VSMCs promotes vascular calcification. We also show that it is mediated by the upregulation of β‐catenin, Runx2, and transcription of osteoblast genes. Furthermore, we found that kinase‐independent function of IKKβ is involved in the regulation of such signaling. Considering that the accelerating roles of NF‐κB in atherosclerosis have been well analyzed, this finding would mechanistically explain the difference between atherosclerosis and calcification.

The difference between atherosclerosis and vascular calcification has been only empirically suggested, but recent clinical studies have clearly demonstrated it.^4^, ^5^, ^6^, ^7^ For instance, Puri et al reported that statins, the most powerful medicine against atherosclerosis, unexpectedly accelerate vascular calcification.^8^, ^35^ However, no mechanistic insights have been reported to explain this finding. Considering statin has the activating effect on β‐catenin^36^ and the suppressive effect on NF‐κB,^12^, ^37^ the observations in this study would account for it, at least in part.

It was also an unexpected observation that more aortic dilatation was seen in KO mice. It has been reported that aortic calcification induces mechanical wall stress by decreasing biomechanical stability, which also associated with destruction and elongation of the elastic lamella in the medial layer.^38^ In addition, mechanical wall stress itself causes expansion and dilatation of the aortic wall.^39^ These mechanisms would account for the dilatation of calcified aorta in KO mice.

The molecular mechanisms of calcification in vessel walls and bone are reported to be similar, as indicated by the observation that the phenotypic transdifferentiation of VSMCs into the osteoblast‐like cells is seen in vascular calcification.^22^, ^23^ Indeed, β‐catenin and Runx2, major regulators of bone‐related genes, such as osterix (*OSX*), alkaline phosphatase (*ALP*), and osteocalcin (*OCN*), promote the transdifferentiation of VSMCs into osteoblast‐like cells.^24^, ^25^, ^26^, ^27^ Therefore, the changes of β‐catenin, Runx2, and osteoblast marker genes seen in KO VSMCs fundamentally support the histological observation of vascular calcification in vivo and in vitro from the molecular level. Furthermore, considering the similarity between bone formation and vascular calcification, it is interesting that previous reports on bone research showed that statin can enhance bone formation.^40^, ^41^, ^42^

Although the roles of β‐catenin in vascular calcification and bone formation have been well analyzed, as previously mentioned, those of IKKβ/NF‐κB signaling have not been adequately unveiled to date. In the bone research, it was reported that the deletion of IKKβ significantly enhanced bone formation by activating β‐catenin ubiquitination and degradation.^43^ As its mechanism, Lamberti et al reported that IKKβ interacts with and is able to phosphorylate β‐catenin,^44^ and Chang et al reported that IKKβ promotes its ubiquitination.^43^ In this study, the nonphosphorylated active form of β‐catenin is suppressed and its ubiquitination is augmented in KD VSMCs. The detailed mechanism of how IKKβ regulates β‐catenin in a kinase‐independent manner is still unclear and is to be unveiled. However, this finding would be of high importance considering that kinase‐independent function of IKKβ has never reported, except by us.^32^ β‐Catenin is the big target of interest, and it has been researched in many fields, including cancer, fibrosis diseases, and vascular calcification.

Considering that kinase‐independent function of IKKβ regulates calcification, as shown in this study, the regulation of protein expression of IKKβ should be more carefully recognized. Interestingly, IKKβ is the target of miR148a, miR503, and miR199a,^45^, ^46^, ^47^ which are upregulated microRNAs in vascular calcification.^48^, ^49^ The suppression of such microRNAs targeting IKKβ might be a new strategy against vascular calcification.

The findings in this study explain not only the difference between atherosclerosis and calcification but also the disappointing results reported in clinical studies that examined the effect of aspirin on calcification. Aspirin has a suppressive effect on inflammatory responses, including NF‐κB. Indeed, Yin et al reported that aspirin is a specific inhibitor of IKKβ.^50^ Therefore, people expect that aspirin use would have a protective effect against calcification; however, recent clinical studies have found that coronary calcification was associated with aspirin use^51^ and that aspirin had no protective effect on the onset of calcification in either coronary arteries or aortic valves. The anticalcification effect of IKKβ revealed in this study might explain these results. Furthermore, we have previously reported that long‐term and high concentration of aspirin can suppress the protein expression of IKKβ,^52^ which might indicate possible modification of kinase‐independent function of IKKβ by aspirin. These new insights can lead to unveiling a new relationship between vascular calcification and aspirin use.

There are strengths and limitations in this study. The strength is that kinase‐independent function of IKKβ in suppressing calcification is unveiled. The kinase‐dependent functions of IKKβ have been well analyzed; however, its kinase‐independent function has been rarely reported, except in our previous study.^32^ Therefore, its roles in diseases are almost unknown. This study indicated its new function in vascular calcification, which would attract interest of researchers in the field of vascular diseases. On the other hand, the limitation of this study is that we have not evaluated the kinase‐dependent roles of IKKβ in the regulation of calcification. Considering that KD‐IKKβ works similarly with KA‐IKKβ in inhibition of calcification, kinase‐independent function would have a major contribution to the inhibitory role of IKKβ on calcification. However, it does not exclude the involvement of kinase‐dependent function of IKKβ on calcification. Some articles have reported that kinase activity of IKKβ regulates β‐catenin.^43^, ^44^, ^53^ Indeed, in Figure 8A, the expression of β‐catenin and Runx2 in KA‐IKKβ cells is less than that in KD‐IKKβ cells, despite less IKKβ protein expression, which implicates the involvement of kinase activity of IKKβ in regulation of these molecules. To explore it precisely, transient expression of constitutive active p65 in vitro would be not suitable because long‐term cell culture is required for observing calcification. Future study of making CaCl_2_ model on mice with modification of p65 or IκB in VSMCs would make it clear.

## Sources of Funding

This work was supported by The Japan Society for the Promotion of Science KAKENHI grants 25461497 and 16H05297, a sponsored research program by Otsuka Pharmaceutical Co Ltd.

## Disclosures

None.

## References

[1] Kovacic JC , Fuster V . Vascular calcification, diabetes, and cardiovascular disease: connecting the dots. JACC Cardiovasc Imaging. 2012;5:367–369. 22498325 10.1016/j.jcmg.2012.02.006 PMC3738006

[2] Block GA , Spiegel DM , Ehrlich J , Mehta R , Lindbergh J , Dreisbach A , Raggi P . Effects of sevelamer and calcium on coronary artery calcification in patients new to hemodialysis. Kidney Int. 2005;68:1815–1824. 16164659 10.1111/j.1523-1755.2005.00600.x

[3] Gassett AJ , Sheppard L , McClelland RL , Olives C , Kronmal R , Blaha MJ , Budoff M , Kaufman JD . Risk factors for long‐term coronary artery calcium progression in the multi‐ethnic study of atherosclerosis. J Am Heart Assoc. 2015;4:e001726 DOI: 10.1161/JAHA.114.001726. 26251281 10.1161/JAHA.114.001726 PMC4599452

[4] Houslay ES , Cowell SJ , Prescott RJ , Reid J , Burton J , Northridge DB , Boon NA , Newby DE ; Scottish Aortic Stenosis and Lipid Lowering Therapy, Impact on Regression trial investigators . Progressive coronary calcification despite intensive lipid‐lowering treatment: a randomised controlled trial. Heart. 2006;92:1207–1212. 16449511 10.1136/hrt.2005.080929 PMC1861190

[5] Terry JG , Carr JJ , Kouba EO , Davis DH , Menon L , Bender K , Chandler ET , Morgan T , Crouse JR III . Effect of simvastatin (80 mg) on coronary and abdominal aortic arterial calcium (from the coronary artery calcification treatment with zocor [CATZ] study). Am J Cardiol. 2007;99:1714–1717. 17560880 10.1016/j.amjcard.2007.01.060

[6] Arad Y , Spadaro LA , Roth M , Newstein D , Guerci AD . Treatment of asymptomatic adults with elevated coronary calcium scores with atorvastatin, vitamin C, and vitamin E: the St Francis Heart Study randomized clinical trial. J Am Coll Cardiol. 2005;46:166–172. 15992652 10.1016/j.jacc.2005.02.089

[7] Raggi P , Davidson M , Callister TQ , Welty FK , Bachmann GA , Hecht H , Rumberger JA . Aggressive versus moderate lipid‐lowering therapy in hypercholesterolemic postmenopausal women: beyond endorsed lipid lowering with EBT scanning (BELLES). Circulation. 2005;112:563–571. 16009795 10.1161/CIRCULATIONAHA.104.512681

[8] Puri R , Nicholls SJ , Shao M , Kataoka Y , Uno K , Kapadia SR , Tuzcu EM , Nissen SE . Impact of statins on serial coronary calcification during atheroma progression and regression. J Am Coll Cardiol. 2015;65:1273–1282. 25835438 10.1016/j.jacc.2015.01.036

[9] Henein MY , Owen A . Statins moderate coronary stenoses but not coronary calcification: results from meta‐analyses. Int J Cardiol. 2011;153:31–35. 20843566 10.1016/j.ijcard.2010.08.031

[10] Kutuk O , Basaga H . Inflammation meets oxidation: NF‐kappaB as a mediator of initial lesion development in atherosclerosis. Trends Mol Med. 2003;9:549–557. 14659470 10.1016/j.molmed.2003.10.007

[11] Pamukcu B , Lip GY , Shantsila E . The nuclear factor—kappa B pathway in atherosclerosis: a potential therapeutic target for atherothrombotic vascular disease. Thromb Res. 2011;128:117–123. 21636112 10.1016/j.thromres.2011.03.025

[12] Antonopoulos AS , Margaritis M , Lee R , Channon K , Antoniades C . Statins as anti‐inflammatory agents in atherogenesis: molecular mechanisms and lessons from the recent clinical trials. Curr Pharm Des. 2012;18:1519–1530. 22364136 10.2174/138161212799504803 PMC3394171

[13] Ortego M , Bustos C , Hernandez‐Presa MA , Tunon J , Diaz C , Hernandez G , Egido J . Atorvastatin reduces NF‐kappaB activation and chemokine expression in vascular smooth muscle cells and mononuclear cells. Atherosclerosis. 1999;147:253–261. 10559511 10.1016/s0021-9150(99)00193-8

[14] Karin M . Nuclear factor‐kappaB in cancer development and progression. Nature. 2006;441:431–436. 16724054 10.1038/nature04870

[15] Perkins ND . Integrating cell‐signalling pathways with NF‐kappaB and ikk function. Nat Rev Mol Cell Biol. 2007;8:49–62. 17183360 10.1038/nrm2083

[16] Trion A , van der Laarse A . Vascular smooth muscle cells and calcification in atherosclerosis. Am Heart J. 2004;147:808–814. 15131535 10.1016/j.ahj.2003.10.047

[17] Basalyga DM , Simionescu DT , Xiong W , Baxter BT , Starcher BC , Vyavahare NR . Elastin degradation and calcification in an abdominal aorta injury model: role of matrix metalloproteinases. Circulation. 2004;110:3480–3487. 15545515 10.1161/01.CIR.0000148367.08413.E9 PMC1262646

[18] Sadekova N , Vallerand D , Guevara E , Lesage F , Girouard H . Carotid calcification in mice: a new model to study the effects of arterial stiffness on the brain. J Am Heart Assoc. 2013;2:e000224 DOI: 10.1161/JAHA.113.000224. 23782921 10.1161/JAHA.113.000224 PMC3698789

[19] Mercurio F , Zhu H , Murray BW , Shevchenko A , Bennett BL , Li J , Young DB , Barbosa M , Mann M , Manning A , Rao A . IKK‐1 and IKK‐2: cytokine‐activated IkappaB kinases essential for NF‐kappaB activation. Science. 1997;278:860–866. 9346484 10.1126/science.278.5339.860

[20] de Carvalho HF , Taboga SR . Fluorescence and confocal laser scanning microscopy imaging of elastic fibers in hematoxylin‐eosin stained sections. Histochem Cell Biol. 1996;106:587–592. 8985747 10.1007/BF02473274

[21] Villa‐Bellosta R , Hamczyk MR . Isolation and culture of aortic smooth muscle cells and in vitro calcification assay. Methods Mol Biol. 2015;1339:119–129. 26445785 10.1007/978-1-4939-2929-0_8

[22] Goettsch C , Hutcheson JD , Aikawa E . Microrna in cardiovascular calcification: focus on targets and extracellular vesicle delivery mechanisms. Circ Res. 2013;112:1073–1084. 23538277 10.1161/CIRCRESAHA.113.300937 PMC3668680

[23] Proudfoot D . Molecular mechanisms of arterial calcification. Artery Res. 2009;3:128–131.

[24] Cai T , Sun D , Duan Y , Wen P , Dai C , Yang J , He W . Wnt/beta‐catenin signaling promotes vsmcs to osteogenic transdifferentiation and calcification through directly modulating runx2 gene expression. Exp Cell Res. 2016;345:206–217. 27321958 10.1016/j.yexcr.2016.06.007

[25] Bostrom KI , Rajamannan NM , Towler DA . The regulation of valvular and vascular sclerosis by osteogenic morphogens. Circ Res. 2011;109:564–577. 21852555 10.1161/CIRCRESAHA.110.234278 PMC3167074

[26] Lin ME , Chen TM , Wallingford MC , Nguyen NB , Yamada S , Sawangmake C , Zhang J , Speer MY , Giachelli CM . Runx2 deletion in smooth muscle cells inhibits vascular osteochondrogenesis and calcification but not atherosclerotic lesion formation. Cardiovasc Res. 2016;112:606–616. 10.1093/cvr/cvw205 PMC5079276 27671804

[27] Lin ME , Chen T , Leaf EM , Speer MY , Giachelli CM . Runx2 expression in smooth muscle cells is required for arterial medial calcification in mice. Am J Pathol. 2015;185:1958–1969. 25987250 10.1016/j.ajpath.2015.03.020 PMC4484217

[28] Stamos JL , Weis WI . The beta‐catenin destruction complex. Cold Spring Harb Perspect Biol. 2013;5:a007898. 23169527 10.1101/cshperspect.a007898 PMC3579403

[29] Proudfoot D , Skepper JN , Hegyi L , Bennett MR , Shanahan CM , Weissberg PL . Apoptosis regulates human vascular calcification in vitro evidence for initiation of vascular calcification by apoptotic bodies. Circ Res. 2000;87:1055–1062. 11090552 10.1161/01.res.87.11.1055

[30] Proudfoot D , Skepper JN , Hegyi L , Farzaneh‐Far A , Shanahan CM , Weissberg PL . The role of apoptosis in the initiation of vascular calcification. Z Kardiol. 2001;90(suppl 3):43–46. 10.1007/s003920170041 11374032

[31] Oeckinghaus A , Hayden MS , Ghosh S . Crosstalk in NF‐kappaB μsignaling pathways. Nat Immunol. 2011;12:695–708. 21772278 10.1038/ni.2065

[32] Ashida N , Senbanerjee S , Kodama S , Foo SY , Coggins M , Spencer JA , Zamiri P , Shen D , Li L , Sciuto T , Dvorak A , Gerszten RE , Lin CP , Karin M , Rosenzweig A . Ikkbeta regulates essential functions of the vascular endothelium through kinase‐dependent and ‐independent pathways. Nat Commun. 2011;2:318–327. 21587235 10.1038/ncomms1317 PMC3113230

[33] Alves RD , Eijken M , van de Peppel J , van Leeuwen JP . Calcifying vascular smooth muscle cells and osteoblasts: independent cell types exhibiting extracellular matrix and biomineralization‐related mimicries. BMC Genom. 2014;15:965. 10.1186/1471-2164-15-965 PMC4247655 25380738

[34] Leopold JA . Vascular calcification: mechanisms of vascular smooth muscle cell calcification. Trends Cardiovasc Med. 2015;25:267–274. 25435520 10.1016/j.tcm.2014.10.021 PMC4414672

[35] Trion A , Schutte‐Bart C , Bax WH , Jukema JW , van der Laarse A . Modulation of calcification of vascular smooth muscle cells in culture by calcium antagonists, statins, and their combination. Mol Cell Biochem. 2008;308:25–33. 17909945 10.1007/s11010-007-9608-1 PMC2226060

[36] Robin NC , Agoston Z , Biechele TL , James RG , Berndt JD , Moon RT . Simvastatin promotes adult hippocampal neurogenesis by enhancing Wnt/beta‐catenin signaling. Stem Cell Reports. 2014;2:9–17. 24511465 10.1016/j.stemcr.2013.11.002 PMC3916759

[37] Jain MK , Ridker PM . Anti‐inflammatory effects of statins: clinical evidence and basic mechanisms. Nat Rev Drug Discovery. 2005;4:977–987. 16341063 10.1038/nrd1901

[38] Li ZY , U‐King‐Im J , Tang TY , Soh E , See TC , Gillard JH . Impact of calcification and intraluminal thrombus on the computed wall stresses of abdominal aortic aneurysm. J Vasc Surg. 2008;47:928–935. 18372154 10.1016/j.jvs.2008.01.006

[39] Speelman L , Bohra A , Bosboom EM , Schurink GW , van de Vosse FN , Makaorun MS , Vorp DA . Effects of wall calcifications in patient‐specific wall stress analyses of abdominal aortic aneurysms. J Biomech Eng. 2007;129:105–109. 17227104 10.1115/1.2401189

[40] Mundy G , Garrett R , Harris S , Chan J , Chen D , Rossini G , Boyce B , Zhao M , Gutierrez G . Stimulation of bone formation in vitro and in rodents by statins. Science. 1999;286:1946–1949. 10583956 10.1126/science.286.5446.1946

[41] Garrett IR , Mundy GR . The role of statins as potential targets for bone formation. Arthritis Res. 2002;4:237–240. 12106493 10.1186/ar413 PMC128929

[42] Wang PSSD , Mogun H , Avorn J . Hmg‐coa reductase inhibitors and the risk of hip fractures in elderly patients. JAMA. 2000;283:3211–3216. 10866868 10.1001/jama.283.24.3211

[43] Chang J , Liu F , Lee M , Wu B , Ting K , Zara JN , Soo C , Al Hezaimi K , Zou W , Chen X , Mooney DJ , Wang CY . NF‐kappaB inhibits osteogenic differentiation of mesenchymal stem cells by promoting beta‐catenin degradation. Proc Natl Acad Sci U S A. 2013;110:9469–9474. 23690607 10.1073/pnas.1300532110 PMC3677422

[44] Lamberti C , Lin KM , Yamamoto Y , Verma U , Verma IM , Byers S , Gaynor RB . Regulation of beta‐catenin function by the IkappaB kinases. J Biol Chem. 2001;276:42276–42286. 11527961 10.1074/jbc.M104227200

[45] Patel V , Carrion K , Hollands A , Hinton A , Gallegos T , Dyo J , Sasik R , Leire E , Hardiman G , Mohamed SA , Nigam S , King CC , Nizet V , Nigam V . The stretch responsive microRNA miR‐148a‐3p is a novel repressor of IKBKB, NF‐kappaB signaling, and inflammatory gene expression in human aortic valve cells. FASEB J. 2015;29:1859–1868. 25630970 10.1096/fj.14-257808 PMC4771066

[46] Yang Y , Liu L , Zhang Y , Guan H , Wu J , Zhu X , Yuan J , Li M . MiR‐503 targets PI3K p85 and IKK‐beta and suppresses progression of non‐small cell lung cancer. Int J Cancer. 2014;135:1531–1542. 24550137 10.1002/ijc.28799

[47] Chen R , Alvero AB , Silasi DA , Kelly MG , Fest S , Visintin I , Leiser A , Schwartz PE , Rutherford T , Mor G . Regulation of IKKbeta by miR‐199a affects NF‐kappaB activity in ovarian cancer cells. Oncogene. 2008;27:4712–4723. 18408758 10.1038/onc.2008.112 PMC3041589

[48] Leopold JA . MicroRNAs regulate vascular medial calcification. Cells. 2014;3:963–980. 25317928 10.3390/cells3040963 PMC4276909

[49] Sun Y , Xu J , Xu L , Zhang J , Chan K , Pan X , Li G . MiR‐503 promotes bone formation in distraction osteogenesis through suppressing Smurf1 expression. Sci Rep. 2017;7:409. 28341855 10.1038/s41598-017-00466-4 PMC5428455

[50] Yin MJY , Yamamoto Y , Gaynor RB . The anti‐inflammatory agents aspirin and salicylate inhibit the activity of I(kappa)B kinase‐beta. Nature. 1998;396:77–80. 9817203 10.1038/23948

[51] Taylor AJ , Bindeman J , Feuerstein I , Le T , Bauer K , Byrd C , Wu H , O'Malley PG . Community‐based provision of statin and aspirin after the detection of coronary artery calcium within a community‐based screening cohort. J Am Coll Cardiol. 2008;51:1337–1341. 18387433 10.1016/j.jacc.2007.11.069

[52] Ashida N , Kishihata M , Tien DN , Kamei K , Kimura T , Yokode M . Aspirin augments the expression of adenomatous polyposis coli protein by suppression of IKKbeta. Biochem Biophys Res Comm. 2014;446:460–464. 24613833 10.1016/j.bbrc.2014.02.134

[53] Sui Y , Park SH , Xu J , Monette S , Helsley RN , Han SS , Zhou C . IKKbeta links vascular inflammation to obesity and atherosclerosis. J Exp Med. 2014;211:869–886. 24799533 10.1084/jem.20131281 PMC4010900

